# Implications of Dietary Guidance for Sport and Exercise

**DOI:** 10.3390/nu15183978

**Published:** 2023-09-14

**Authors:** Julio Calleja-González

**Affiliations:** 1 Department of Physical Education and Sports, Faculty of Education and Sport, University of the Basque Country, (UPV/EHU), 01007 Vitoria-Gasteiz, Spain; julio.calleja.gonzalez@gmail.com; 2 Faculty of Kinesiology, University of Zagreb, 10110 Zagreb, Croatia

The importance of nutrition in maintaining health and improving sports performance is well known. Each sports modality requires a specific intake of food and supplements. In addition, nutritional timing, hydration, diet, and nutritional supplementation are key to increasing athletes’ performance. Further, nutritional strategies can also allow the recovery period to be shorter, which can lead to a higher training and competition load. However, athletes often suffer from gastrointestinal disturbances due to high pressure to win, long-distance travel, or the nutritional disorders they experience to meet their specific demands. In this context, it is important to focus not only on the athletes’ performance but also on their health and recovery. This Special Issue highlights potential and present future nutritional strategies to improve health, sports performance and recovery after exercise.

Initially it presents three systematic reviews. The first one meta-analyzed the effects of probiotic supplementation on exercise in order to improve performance in a test in which aerobic metabolism is predominant in the trained population. However, more research is needed to fully understand the mechanisms of action of this supplement. The results suggest that greater benefits may be obtained when the duration of supplementation is ≤4 weeks with a single strain or when the dose of supplementation is ≥30 × 10^9^ colony forming units. In addition, the probiotics seem to more effective in men and in tests performed to exhaustion [1]. The second review concluded that oral -L-Carnitine supplementation with 3 to 4 g ingested between 60 and 90 min before testing or 2 to 2.72 g/day for 9 to 24 weeks improved high-intensity exercise performance. Nevertheless, chronic or acute oral L-Carnitines or glycine-propionyl L-Carnitine supplementation did not present improvements in moderate exercise performance [2]. Equally, the third one in the basketball field, the effective dose of caffeine to enhance anaerobic performance and the feeling of vigorousness and energy ranges from 3 to 6 mg·kg^−1^, showing more positive effects when supplemented 60–75 min before exercise in the morning and in test-based tasks. To improve recovery and wellness, some nutritional supplements may have promising benefits for basketball. This is the case of Vitamin E (ranging from 200 to 268 mg during 4–6 weeks), vitamin D (10,000 IU/day) and EPA (2 g during 6 weeks). [3].

Further, this Special Issue proposes a transversal, cross-sectional, observational, and descriptive study in basketball with 104 participants who described that the knowledge of sport-specific nutrition in players under 18 years old, as well as non-professional and professional adult basketball players, is insufficient through all the categories and levels [4]. Finally, the other cluster study suggests that pro-healthy dietary patterns and lower mileage may favor higher bone mineral density in male amateur marathoners [5].

This Issue also includes randomized control trials. The first one analyzed supplementation with 3 g/day of Citrulline and 2.1 g/day of beetroot extract (300 mg/day of NO_3_^−^) for 9 weeks on maximal and endurance strength, presenting improvements in performance aerobic power tests [6]. 

On the other hand, the wheelchair basketball game in hot leads to significantly higher sweat rate and loss in body mass compared to temperate conditions. Even relative body mass loss was less than 2%, players thus have to be supported with enough fluid, especially during games in hot environments [7]. Lastly, a study featured a 17-week follow-up on the effects of individual and group nutrition intervention on changes in eating habits and selected biochemical parameters in young soccer players. The teenagers from the latter group consumed less saccharose (44 g vs. 39.2 g) in favor of digestible CHO (266 g vs. 273 g) and dietary fiber (19.7 g vs. 22.2 g), further emphasizing the health-promoting profile of diets. The amount of fluid consumed (33% vs. 48% above 2 L of water a day) and the habits of the peri-workout hydration routine were also improved. Many of the participants (41%) reported faster regeneration while 26% experienced an overall better well-being [8]

The diversity of articles published in this Special Issue, “Implications of Dietary Guidance for Sport and Exercise”, describes the role of ergogenic aids, nutrients, and dietary supplements on a variety of sport performance models.

Future studies should analyze these conclusions featuring other sports populations.

## References

[1] Santibañez-Gutiérrez A., Fernández-Landa J., Calleja-González J., Delextrat A., Mielgo-Ayuso J. (2022). Effects of Probiotic Supplementation on Exercise with Predominance of Aerobic Metabolism in Trained Population: A Systematic Review, Meta-Analysis and Meta-Regression. Nutrients.

[2] Mielgo-Ayuso J., Pietrantonio L., Viribay A., Calleja-González J., González-Bernal J., Fernández-Lázaro D. (2021). Effect of Acute and Chronic Oral l-Carnitine Supplementation on Exercise Performance Based on the Exercise Intensity: A Systematic Review. Nutrients.

[3] Escribano-Ott I., Calleja-González J., Mielgo-Ayuso J. (2022). Ergo-Nutritional Intervention in Basketball: A Systematic Review. Nutrients.

[4] Escribano-Ott I., Mielgo-Ayuso J., Calleja-González J. (2021). A Glimpse of the Sports Nutrition Awareness in Spanish Basketball Players. Nutrients.

[5] Bykowska-Derda A., Zielińska-Dawidziak M., Czlapka-Matyasik M. (2022). Dietary-Lifestyle Patterns Associated with Bone Turnover Markers, and Bone Mineral Density in Adult Male Distance Amateur Runners-A Cross-Sectional Study. Nutrients.

[6] Burgos J., Viribay A., Fernández-Lázaro D., Calleja-González J., González-Santos J., Mielgo-Ayuso J. (2021). Combined Effects of Citrulline Plus Nitrate-Rich Beetroot Extract Co-Supplementation on Maximal and Endurance-Strength and Aerobic Power in Trained Male Triathletes: A Randomized Double-Blind, Placebo-Controlled Trial. Nutrients.

[7] Grossmann F., Perret C., Roelands B., Meeusen R., Leonie Flueck J. (2022). Fluid Balance and Thermoregulatory Responses during Wheelchair Basketball Games in Hot vs. Temperate Conditions. Nutrients.

[8] Grabia M., Markiewicz-Żukowska R., Bielecka J., Puścion-Jakubik A., Socha K. (2022). Effects of Dietary Intervention and Education on Selected Biochemical Parameters and Nutritional Habits of Young Soccer Players. Nutrients.

